# Mathematical model of aging in COVID-19

**DOI:** 10.5937/jomb0-39602

**Published:** 2023-08-25

**Authors:** Olivera Jovanikić, G. Stevanović, Boban Đorđevic, Milan Jovanović, Milan Lepić

**Affiliations:** 1 University of Defense, Faculty of Medicine, Belgrade; 2 Clinic for Neurosurgery, MMA, Belgrade; 3 University of Belgrade, Faculty of Medicine, Belgrade; 4 Institute for Infectious and Tropical Diseases, Belgrade; 5 Clinic for Plastic and Reconstructive Surgery, MMA, Belgrade; 6 Clinic for Abdominal and Endocrine Surgery, MMA, Belgrade

**Keywords:** COVID-19 virus, mathematical model, intima-media thickness, carotid. C-reactive protein, interleukin-6, fibrinogen, ultrasonography, pneumonia, COVID-19 virus, matematički model, debljina intime-medija, karotida, C-reaktivni protein, interleukin-6, fibrinogen, ultrazvuk, pneumonija

## Abstract

**Background:**

The aim was examination of the intimamedia thickness of carotid arteries in COVID-19 infection.

**Methods:**

In 50 patients, the thickness of the intimomedial complex (IMT) in the common carotid arteries was measured. The values were compared with the control group in 2006-9. The condition of the lungs was assessed by ultrasound score (It score) (0-42) as mild (0-14) or mediumsevere (15-28) Covid. IMT thickening risk factors and the value of fibrinogen, IL-6 and CRP were recorded. Two IMT prediction models were formed. The socio-epidemiological model predicts the development of IMT based on epidemiological factors. Apart from these factors, the second model also includes the values of the mentioned biomarkers.

**Results:**

It score 20±6, IMT values right: median 0.99 mm, p25=0.89, p75=1.14; left: 1±0.22 mm. Control: IMTright: median 0.7 mm, p25=0.68 mm; p75=0-9 mm; left: median=0.75 mm, p25=0.6 mm, p75=1.0 mm. The group/control difference is highly significant. Epide mio - logical model: logit (IMT)= 4.463+(2.021+value for GEN)+(0.055x AGE value)+(-3.419x RF value)+(-4.447x SM value)+(5.115x HTA value)+(3.56x DM value)+ (22.389x LIP value)+(24.206x CVD value)+(1.449x other value)+(-0.138x It score value)+(0.19xBMI value). Epidemiological-inflammatory model: logit (IMT)=5.204+ (2.545x GEN value)+(0.076x AGE value)+(-6.132x RF value)+(-7.583x SM value)+(8.744x HTA value)+(6.838x DM value)+(25.446x LIP value)+(28.825x CVD value)+ (2.487x other value)+(-0.218xIt score value)+(0.649x BMI value) +(-0.194x fibrinogen value)+(0.894x IL-6 value)+(0.659x CRP value). Values for both models Exp(B)=4.882; P of sample=0.83; logit=-0.19; OR= 23.84; model accuracy for the first model 87% and for the second 88%; Omnibus test of the first model c2=34.324; p=0.000; reliability coefficient -2LogLH=56.854; Omnibus test of the second model c2=39.774; p=0.000; and -2LogLH=51.403.

**Conclusions:**

The ageing of blood vessels in COVID-19 can be predicted.

## Abstract (sr)

**Uvod:**

Cilj je bio ispitivanje debljine intimomedijalnog kompleksa karotidnih arterija u COVID-19 infekciji.

**Metode:**

Kod 50 pacijenata sa PCR(+) testom merena je debljina intimomedijalnog kompleksa (IMT) u zajedničkim karotidnim arterijama. Vrednosti su upoređene sa kontrolnom grupom 2006-9 God. Stanje pluća ocenjeno je ultrasonografskim skorom (It skor) (0-42) lak (0-14) ili srednje težak COVID-19 (15-28). Beleženi su riziko faktori zadebljanja IMT i vrednost fibrinogena, IL-6 i CRP. Formirana su dva modela predviđanja IMT. Socio-epidemiološki model predviđa razvoj IMT na osnovu epidemioloških faktora. Drugi model, osim ovih faktora uključuje i vrednosti navedenih biomarkera.

**Rezultati:**

It skor pluća 20±6 i vrednosti IMT desno mediana 0,99 mm, p25=0,89 mm, p75=1,14 mm i levo: medijana = 0,75 mm, p25=0,6 mm, p75=1.0 mm. Kontrolna grupa IMT desno mediana 0,7 mm, p25= 0,68 mm, p75=0,9 mm i levo mediana 0,75 mm, p25=0,6 mm, p0,75=1 mm (nema razlike strana p=0,864). Razlika grupa/kontrola je visoko značajna (p=0,000). Epidemiološki model: logit (IMT)=4,463+(2,021+vrednost za GEN)+(0,055x vrednost za AGE)+(-3.419x vrednost za RF)+(-4.447x vrednost za SM)+(5,115x vrednost za HTA)+(3,56x vrednost za DM)+(22,389x vrednost za LIP)+(24,206x vrednost za KVO)+(1,449x vrednost za drugo)+(-0,138x vrednost za It skor)+(0,19x vrednost za BMI). Epidemiološko-inflamatorni model: logit(IMT)= 5,204+(2,545x vrednost GEN)+(0,076x vrednost za AGE)+(-6,132x vrednost za RF)+(-7,583x vrednost za SM)+(8,744x vrednost za HTA)+(6,838x vrednost za DM)+(25,446x vrednost za LIP) + (28,825x vrednost za KVO)+(2,487 x vrednost za drugo)+(-0,218x vrednost za It skor)+ (0,649x vrednost za BMI)+(-0,194x vrednost za fibrinogen)+(0,894x vrednost za IL-6) + (0,659x vrednost za CRP). Vrednosti za oba modela Exp(B)=4,882; P uzorka=0,83; logit=-0,19; OR=23,84; preciznost modela 87% i 88% (prvi, drugi model, redom); Omnibus test prvog modela c2=34,324; p=0,000; koeficijent verodostojnosti -2LogLH=56,854; Omnibus test drugog modela c2=39,774; p=0,000; -2LogLH= 51,403.

**Zaključak:**

Starenje krvnih sudova u COVID-19 se može predvideti.

## Introduction

Every year, the intima-media thickness of the common carotid arteries (cIMT) increases by 0–40 μm [1]. The layer of endothelial cells is thin and cannot be measured or shown in isolation but only together with the media layer of the blood vessel - the intima-media complex [2]. This structure can only be seen on longitudinal B-mode ultrasound imaging of arteries that are immobile, superficial, and straight-flow [2] [3]. Normal values and a detailed IMT examination protocol are best defined for the distal part of the common carotid arteries [2] [3].

Ageing – spontaneous thickening of cIMT can be accelerated under the influence of well-known factors: hyperlipidemia, diabetes, obesity, smoking, gender, hypertension, systemic diseases, and genetic factors [1] [4] [5] [6] [7]. Modern researchers believe that cIMT thickening is greater than expected for age, actually an injury of the intimomedial layer or arteriopathy [8] [9]. The influence of infectious agents on IMT is the least studied, but it is known: Chlamydia pneumoniae, cytomegalovirus, H. simplex virus type 1 and 2 and HIV cause the thickening of IMT [5] [10] [11] [12]. Their introduction into certain strains of experimental animals by inhalation into the nose or trachea after 30 days causes histological changes in the blood vessels that correspond to the first steps in the development of atherosclerosis [5] [13] [14].

On the other hand, 1/3 of lung cells are endothelial cells, and ARDS that occurs in a COVID- 19 infection also represents the dysfunction and activation of lung endothelial cells [15] [16]. The condition of the lungs under COVID-19 can be quantified by the US lung score – It score [17]. Endothelial cells are the first line of defence in the inflammatory response to tissue damage or microbial infection [18]. Numerous proteins and their receptors (pattern recognition receptors PRRs) participate in this response, which also participates in the process of atherosclerosis [19]. Dysfunction and activation of the endothelium often lead to hypercoagulation [20] and thrombosis [21] [22], which is often observed in COVID-19 [20] [21] [22]. One of the main proteins of endothelial dysfunction and activation is CRP [23] [24]: its high level in the blood indicates a poor prognosis in the course of COVID-19 [24]. Research links CRP level with damage and severe dysfunction of the endothelium through activation of pro-inflammatory genes in endotheliocytes and p38-mediated apoptosis [25]. Examination of the patient’s coagulation status often shows high fibrinogen values, increased platelet activation, and increased plasma viscosity in severe cases of COVID-19 [26] [27]. Elevated values of IL-6 were also noted in COVID-19 [28] [29]. This biomarker is produced by damaged (infected) endo - theliocytes, monocytes/macrophages, and adipocytes [30]. A high level of IL-6 leads to increased permeability of the endothelium, leucocytes recruitment and infiltration of the vessels wall [31]. The increase in oxidative stress level and the vascular spasm (reduced a NO bioavailability) is also caused by the increased level of IL-6 in the blood [31].

There is a basis for examining cIMT change at a typical site and its relationships with epidemiological risk factors and biochemical markers of endothelial dysfunction and activation in COVID-19.

## Materials and methods

From October 2019 to April 2020, a prospective, randomized cohort cross-sectional study of cIMT thickness in patients with COVID-19 was conducted at the Institute. Permission from the local ethics committee to conduct the research was obtained. All patients had a positive PCR test for COVID-19 upon admission. Lung ultrasound examination was performed with a liner probe (PLT-1204BT) in the program for carotid arteries with resolution 7–14 MHz, on the device JP, Toshiba Aplio 500 & Overbar, Tawara Tochi, Japan.

The lung ultrasound examination protocol and evaluation (It score) were performed according to Soldati G. et al. [17]. We divided the It score values into light (0–14), medium (15–28) and severe COVID-19 (29–42). In 50 consecutive patients with a mild/moderate US lung score, the IMT thickness of the posterior wall of the common carotid arteries was measured [1] [2].

IMT was measured on both sides, in the most distal part of the common carotid artery (segment up to 10 mm long), with no plaque on the far wall [1] [2]. The measurement was done following the Manheim consensus [3]: with a linear probe (7–14 MHz) at a focus depth of 30–40 mm, to obtain a symmetrical brightness on the near and far wall. The gain settings, the frame rate, and log gain compensation are set on the device, so that intraluminal artefacts are minimal. IMT values were obtained as the mean value from three measurements (1–3). All IMT values are marked as 0 – normal (up to 900 μm), 1 – elevated (>900 μm) [1].

During our research in the period 2004–2009, the value of IMT was measured in the same way in 50 subjects, in whom all studied risk factors were excluded by additional tests, recordings and analyses [32]. Also, they were not exposed to the COVID-19 infection at the time.

The IMT values measured from the right and left sides in the patient group did not differ significantly (Mann-Whitney U test: z=1305; p=0.704), so 100 IMT values were considered as one data group. IMT values in the control group on the right and left sides did not differ significantly (Mann-Whitney U test: z=1264, p=0.922), so 100 IMT values were considered as one data group.

The IMT values of the whole group (100) were compared with the IMT values of the control group (100) (Mann-Whitney U test).

In the research, we noted the existence of risk factors that affect cIMT: hypertension (HTA), smoking (SM), body mass index (BMI), diabetes mellitus(DM), hyperlipidemia (Lip), other cardiovascular diseases (KVD), as well as all conditions (marked as others) that lead to an increase in WSS (wall shear stress) and possible endothelial damage (malignancies, systemic diseases, radiotherapy, chemotherapy, thrombophilias). We classified the total number of listed risk factors as 0 – no factor, 1 – one risk factor, 2 – two or more risk factors.

Body mass index was calculated according to the formula: TT/TV2 (TT – body weight in kg, TV – body height in m) and classified: 0 – normal value (18.5–24.9); 1 – underweight and overweight (below 18.5, 25–29.9 respectively); 2 – obesity (30 and above) [33].

In all patients, the value of fibrinogen (1.8–3.5 g/L), interleukin-6 (IL-6) (0–7 pg/L), and C-reactive protein (CRP) (0–3 mg/L) was determined. We classified the obtained results: fibrinogen 0.1 and 2 (<3.5; 3.5–6; >6 respectively), IL-6 0.1.2 and 3 (0– 7; >7–50; >50–100; >100 respectively), CRP 0, 1, 2 (0–50; 50–100; 100 respectively).The examined group was divided into three subgroups: 45 years, 46–65 and >65 years. After examining the normality of the distribution, the significance of the difference in IMT values between the 45 years and >65 years group was tested (Mann-Whitney test) (Table 1).

**Table 1:** Classification of risk factors and biomarkers in research.

Classification	Risk factors	BMI kg/m^2^	Fibrinogen g/L	IL-6 pg/mL	CRP
0	without risk factors	18.5–24.9 normal values	<3.5	0-7	0-50
1	one risk factor	<18.5 or 25–25.9 underweight <br>or overweight	3.5-6	>7-50	≥50–100
2	two or more risk factors	≥30 obesity	>6	>50-100	≥100
3	-	-	-	>100	-

Linear logistic regression excludes a strong linear relationship between the predictor variables.

Based on the obtained data, two binary logistic models for predicting the development of IMT enlargement (900–1500 μm) were formed.

**The first – epidemiological model** predicts the development of IMT thickening based on the number and type of risk factors and It score (US lung examination).

**The second epidemiological – is inflammatory**, in which, in addition to the number and type of risk factors for thickening of IMT, It score, inflammatory markers fibrinogen, CRP and IL-6 are also included. Both models allow the chance and probability of IMT thickening to be determined in a specific patient with COVID-19 infection.

## Results

In the examined group of patients with COVID- 19 in whom the IMT value was measured, the average age was 54±14 (20–82), CV=26%. There were 26 women (52%) and 24 men (48%); the difference in the number of men and women is not significant ( 2=0.08; p=0.77). The average duration of symptoms before examination and examination was 38 days (2–119 days), with a median of 18 days.

The average value of the It score was 20±6; CV=30%; 18% (9) had a mild finding, and 82% (41) had a medium-severe finding.

Among all measured IMT values, 83% were in the range of 900–1500 μm. The median IMT value on the right side was 0.99 mm, p_25_=0.89 mm; p_75_=1.14 mm (0.6–1.5 mm) and on the left side, 1±0.22 mm (0.49–1.5 mm). It was determined that there was no difference in the data from the right and left sides (Mann-Whitney test, p=0.704), and all measurements were combined into one group (median 1.0 mm, p_25_=0.9 mm, p_75_=1.10 mm).

Subgroups of patients aged 45 (14 patients) and over 65 (12 patients) were formed. IMT values in these subgroups did not differ significantly (Mann- Whitney test, p=0.102).

The average value of IMT in the control group (50 subjects) from the period 2004–2009 was a median of 0.7 mm, p_25_= 0.68 mm and p_75_=0.9 mm on the right and a median 0.75 mm, p_25_=0.6 mm, p_75_=1.0 mm on the left, but the values were combined because the differences are not significant (Mann-Whitney U test, p=0.922). The group did not differ from the control in terms of age (Mann-Whitney U test, p=0.772) and the ratio of women to men ( 2=1.423; p=0.233). Testing the significance of the difference in IMT values in the group and the control, we found that the difference is highly significant (Mann-Whitney test, p=0.000).

The distribution of epidemiological risk factors for IMT increase is given in (Table 2). The number of patients is indicated, and the percentage of patients in the group is shown in parentheses.

**Table 2:** Sociodemografic characteristics of group and control and mean values of the IMT and It score. Dates are presented as mean ± SD, or the median, p25, p75

	Age, y	Gender <br>prevalence of <br>men, n (%)	IMT values <br>on the right <br>(mm) <br>N=50	IMT values <br>on the left <br>(mm) <br>N=50	IMT values <br>on both sides <br>(mm) <br>N=100	the total <br>number of <br>elevated <br>IMT values, <br>N=100, n (%)	It score <br>N=50, <br>n (%)
Group	54±14	26(52)	0.99 <br>p_25_=0.89 <br>p_75_=1.14	1±0.22	1.0 <br>p_25_=0.9 <br>p_75_=1.1	83 (83%)	20±6 <br>9(18) – <br>mild, <br>41(82) – <br>medium <br>severe
Control	52±18	29(58)	0.7 <br>p_25_=0.68 <br>p_75_=0.9	0.75 <br>p_25_=0.6 <br>p_75_=1.0	0.78±0.25	34 (34%)	–

Strong collinearity between the selected predictors (gender, age, smoking, hypertension, diabetes, lipid values, It score, BMI, presence of cardiovascular and other diseases) is excluded by linear logistic regression: tolerance in no case is less than 0.1; VIF values are not greater than 10 for any predictor variable of the model. Mahalanobis statistics conditions for df=9 also rule out a solid linear connection between the predictors. Intercorrelations between predictors are also excluded: no correlation coefficient is more significant than 0.7.

Sample chance = 4.882; Sample probability 0.83; Logit: log odds (LO)=-0.19; Odds ratio (OR)=23.84; Accuracy of the model with selected predictors 87%; Omnibus test 2= 34,324; df=11; p=0.000; reliability coefficient -2LogLH= 56.854.

To form the epidemiological-inflammatory binary model, strong collinearity between the selected predictors was first excluded, with linear logistic regression: tolerance is not lower than 0.1, and VIF values are not higher than 10 for any model variable. Mahalanobis statistics conditions for df=12 also exclude strong linear correlation between predictors, Intercorrelations between predictors are also excluded: no correlation coefficient is greater than 0.7.

Sample chance: exp(B)=4.882; Sample probability: 0.83; log odds (LO):-0.19; Odds ratio OR=23.84; accuracy of the model with selected predictors 88%; Omnibus test: 2=39.774; p=0.000; df=14; Reliability coefficient: -2LogLH= 51.403.

## Discussion

In our research on the COVID-19 infection, we tried to have an original and completely different approach to the most critical aspect of this problem – changes in the endothelium of blood vessels. The modern resolution of ultrasound machines is a tenth of a mm, and the mean value of IMT from our three measurements allows the data to be sound and well-controlled. The well-known data on the rate of increase in IMT value during ageing [1] enables the assessment of the objective age of the common carotid artery by examining it with ultrasonography in B-mode. Moreover, this method is easy to implement and repeatable.

The IMT values in our research and in our control group (from the 2004-9 research) show a highly significant difference, although the subjects did not differ in age or gender [32]. At that time, there was no infection with the SARS-CoV2 virus.

Comparison of IMT values within the group with COVID-19 in the subgroup of those younger than 46 and older than 64 shows a distinct ageing of IMT (the difference in IMT values is not significant p=0.102 >0.05).

In experiments in which changes in blood vessels are observed after infection with an infectious agent, it is stated that the changes develop after a certain number of days [5]. In our research, the period from the appearance of the first symptoms to the examination of the arteries by ultrasound was most often 18 days (38 days on average). There was enough time to develop a noticeable senescence-thickening of the IMT. Whether the process of IMT thickening still continues even after negative PCR tests and clinical cure will be revealed by future research. In the studied group, men have a several times higher risk of IMT thickening (7.6 and 12.7 times). This may indicate a protective role of estrogen in the virus-induced thickening of IMT [1]. The group’s most common risk factors for IMT thickening were smoking (76%) and hypertension (40%). Hyperlipidemia and diabetes, which damage the artery endothelium the most, were present in only 8% and 12%. Regardless, as many as 83% of patients have elevated IMT values. Fibrinogen and CRP values were elevated in 79% and 70% of patients, respectively, and these were mostly moderately elevated values (Table 3). As in many studies so far, this finding reflects dysfunction and intensive activation of the endothelium ([23] [24] [30] [31]).

**Table 3:** Stratification of the epidemiological risk factors and their numbers.

Degree	0	1	2	3	total
Number of risk factors	20(40%)	15(30%)	15(30%)	/	50(100%)
Smoking	12(24%)	38(76%)	/	/	50(100%)
Diabetes mellitus	44(88%)	6(12%)	/	/	50(100%)
Hypertension	30(60%)	20(40%)	/	/	50(100%)
Hyperlipidemia	46(92%)	4(8%)	/	/	50(100%)
Other KVD	45(90%)	5(10%)	/	/	50(100%)
BMI	11(22%)	18(36%)	15(30%	6(12%)	50(100%)
Other diseases	38(76%)	12(24%)	/	/	50(100%)

Binary logistic regression models can predict the development of IMT thickening in COVID-19. A large number of predictors is rarely used in regression models because it is difficult to avoid a strong linear relationship between the predictors. However, such models allow conclusions to be drawn on a smaller sample as if it were a many times larger number of study participants. Both models use simple predictor variables that are not difficult to record during a pandemic. Both models make sense to apply to the population with mild/moderate COVID-19: Omnibus test in the first model χ^2^=34.324; p=0.000; and in the second χ^2^=39.774; p=0.000. Both models successfully (-2LogLH=56.854; and -2LogLH= 51.403) and accurately (87% and 88%) can help predict the increase in IMT in patients with mild and moderate COVID-19 based on the selected predictors.

In the first model, the predictor variables that significantly influence the increase in IMT are gender, smoking, hypertension, and one can also say It score (p is 0.035, 0.021, 0.03, and 0.055, respectively). Among all risk factors for the development of IMT thickening, hypertension has the greatest impact and increases the chance of IMT thickening 166.4 times. Men, compared to women, have a 7.6 times greater chance of developing an increase in IMT, while the influence of smoking (0.012 times) and It score values (0.871 times) is negligibly small.

In the second model, biomarker values of endothelial dysfunction (fibrinogen, CRP and IL-6) were added to the predictor variables. In this model, the development of IMT thickening is significantly influenced by gender, the number of risk factors, smoking, hypertension, It score, and CRP level (p is 0.038; 0.046; 0.013; 0.028; 0.05; 0.05, respectively). Hypertensive patients have the highest chance of IMT thickening in COVID-19, as much as 6272 times higher than those without hypertension. Men are 12.7 times more likely to develop IMT thickening than women. An increase in the value of CRP by 50 mg/L in the blood carries almost twice the risk for thickening IMT. The impact of changing the value of the It score, smoking and the number of risk factors by 1 is negligibly small: 0.804, 0.001, and 0.002 times, respectively (Table 4, Table 5, Table 6, Table 7).

**Table 4:** Clinical markers tested in the group, its values and IMT values.

Variables	Control value	Group	Control
fibrinogen	1.8–3.5 g/L	2.1–11.5 g/L	-
IL-6	0.7 pg/mL	5.3–727.6 pg/mL	-
CRP	0–3 mg/L	1.7–206.0 mg/L	-
IMT values on both sides	<900 mm	0.49–1.5 mm	0.4–1.5 mm
IMT values on the right	<900 mm	0.6–1.5 mm	0.4–1.3 mm
IMT values on the left	<900 mm	0.49–1.5 mm	0.4–1.5 mm

**Table 5:** Biomarkers of endothelial activation and dysfunction in the examined group (percentage of patients with that biomarker value and their range in brackets).

Marker	0	1	2	3
Fibrinogen	21% (<3.5)	55% (3.5–6)	24% (>6)	/
CRP	30% (0–49)	48% (50–99)	22% ( 100)	/
IL-6	2% (0–7)	30% (8–50)	44% (51100)	24% (>100)

**Table 6:** Epidemiological binary logistic regression model predicting ageing (thickening of IMT) in COVID-19 (IMT is 0 for values up to 900 μm, and 1 for 900–1500 μm).

Variables in the Equation	Epidemiological binary regression model				
	B	Sig.	Exp(B)	95% C.I.for EXP(B)	
				Lower	Upper
GENDER	2.021	0.035	7.549	1.158	49.224
AGE	0.055	0.076	1.056	0.994	1.122
RISK FACTORS	-3.419	0.056	0.033	0.001	1.084
SMOKING	-4.447	0.021	0.012	0.000	0.517
HTA	5.115	0.030	166.454	1.619	17111.547
DM	3.650	0.213	38.457	0.123	12001.248
LIP	22.389	0.999	5288321164. 113	0.000	-
KVD	24.206	0.998	3253744406.076	0.000	-
OTHERS	1.449	0.254	4.257	0.354	51.175
ITSCOR	-0.138	0.055	0.871	0.756	1.003
BMI	0.190	0.649	1.210	0.533	2.746
Constant	4.463	0.138	86.736		

**Table 7:** Epidemiological-inflammatory binary logistic regression model predicting ageing (thickening of IMT) in COVID-19. <br>IMT is 0 for values up to 900 μm, and 1 for 900–1500 μm.

Variables in the Equation		Epidemiological-inflammatory binary logistic regression				
		B	Sig.	Exp(B)	95% C.I.for EXP(B)	
					Lower	Upper
Step 1a	GENDER	2.545	0.038	12.741	1.153	140.759
	AGE	0.076	0.066	1.079	0.995	1.169
	RISK FACTORS	-6.132	0046	0.002	0.000	0.886
	SMOKING	-7.583	0.013	0.001	0.000	0.201
	HTA	8.744	0.028	6272.306	2.556	15394500.283
	DM	6.838	0.109	932.731	0.220	3950930.413
	LIP	25.446	0.998	112437365184.723	0.000	-
	KVD	28.825	0.998	3300217867627.873	0.000	-
	OTHERS	2.487	0.221	12.030	0.224	645.860
	ITSCOR	-0.218	0.050	0.804	0.646	1.000
	BMI	0.649	0.260	1.913	0.618	5.923
	fibrinogen	-0.194	0.726	0.823	0.277	2.446
	ILE6	0.894	0.238	2.444	0.554	10.773
	CRP	0.659	0.050	1.933	1.001	3.731
	Constant	5.204	0.182	181.939		

Both models can be applied to a specific patient.

IMT thickening is an expected ageing process that is accelerated by the infection of COVID-19 and the effect of the above factors. The question of applying previous experience in the prevention and treatment of IMT thickening and in treatment of IMT thickening in COVID-19 is opened. Further research is needed.

## Dodatak

### List of abbreviations:

IMT, thickening of the intima-media complex;<br>GEN, gender;<br>AGE, years;<br>RF, number of risk factors; <br>DM, diabetes;<br>SM, smoking;<br>HTA, hypertension;<br>LIP, lipidemia;<br>CVD, cardiovascular diseases;<br>other value, other diseases or condition important for IMT;<br>BMI, body mass index;<br>It score, ultrasound score of the lung ultrasonography

### Conflict of interest statement

All the authors declare that they have no conflict of interest in this work.
